# Ingroup/outgroup membership modulates fairness consideration: neural signatures from ERPs and EEG oscillations

**DOI:** 10.1038/srep39827

**Published:** 2017-01-04

**Authors:** Yiwen Wang, Zhen Zhang, Liying Bai, Chongde Lin, Roman Osinsky, Johannes Hewig

**Affiliations:** 1 School of Humanities and Social Sciences, Institute of Psychological and Cognitive Sciences, Fuzhou University, Fuzhou, 350116, China; 2 Institute of Developmental Psychology, Beijing Normal University, 100875, China; 3 Department of Psychology, University of Osnabrück, 49074 Osnabrück, Germany; 4 Department of Psychology, Julius-Maximilians-University Würzburg, 97070, Würzburg, Germany

## Abstract

Previous studies have shown that ingroup/outgroup membership influences individual’s fairness considerations. However, it is not clear yet how group membership influences brain activity when a recipient evaluates the fairness of asset distribution. In this study, subjects participated as recipients in an Ultimatum Game with alleged members of both an experimentally induced ingroup and outgroup. They either received extremely unequal, moderately unequal, or equal offers from proposers while electroencephalogram was recorded. Behavioral results showed that the acceptance rates for unequal offers were higher when interacting with ingroup partners than with outgroup partners. Analyses of event related potentials revealed that proposers’ group membership modulated offer evaluation at earlier processing stages. Feedback-related negativity was more negative for extremely and moderately unequal offers compared to equal offers in the ingroup interaction whereas it did not show differential responses to different offers in the outgroup interaction. Analyses of event related oscillations revealed that the theta power (4–6 Hz) was larger for moderately unequal offers than equal offers in the ingroup interaction whereas it did not show differential responses to different offers in the outgroup interaction. Thus, early mechanisms of fairness evaluation are strongly modulated by the ingroup/outgroup membership of the interaction partner.

Fairness considerations, i.e. comparisons of self-interest and other-interest, are a strong motivational force in social interactions^1^. Behavioral research has demonstrated that individuals are not purely rational beings aiming to maximize self-gain but also care about their relative benefits in comparison to others^2^,^3^. One way to investigate fairness considerations in asset division is to let individuals play economic exchange games, like the Ultimatum Game (UG)^4^. The UG is a widely used scenario where a proposer offers to divide money between herself/himself and another player. When the recipient accepts, both gain. When he/she does not accept, neither person receives anything. Recipients typically reject offers of 20% of the total sum about half of the time, and rejection rates increase as recipient shares become smaller^2^,^3^,^4^. Thus, rejection rates in the UG reflect the recipient’s considerations of which offer is fair and which is unfair. Alternatively, given that rejecting unfair offer means punishing selfish proposers at a personal cost, some researchers also describe the rejection as altruistic punishment^5^ or costly punishment^6^.

A social factor which seems to influence such fairness considerations is the group membership of the interaction partners. A growing body of studies have explored the group bias in fairness norm enforcement, but these results are inconsistent^7^,^8^,^9^,^10^,^11^. Some authors propose a Norm-Focused Theory, emphasize the functional role of prescriptive norms which help to maintain group cohesion and promote group interest. Accordingly, the violation of such norms will be highly salient and objectionable, violating the central compact of group life^12^. According to the Social Identity Theory, other researchers propose that an ingroup member’s unfair action will at least partially be compensated by the positive evaluation they gain through group membership^13^,^14^. Hence, these two theories make different predictions about punishment behaviors in intragroup interaction relative to intergroup interaction. The Norm-Focus Theory predicts that ingroup norm violators are punished more harshly than outgroup norm violators, while the Social Identity Theory predicts that ingroup norm violators are punished less harshly than outgroup norm violators. In the Ultimatum Game, both theories have been supported. A recent review of the literature appraised studies on group bias in norm enforcement and indicated that most findings are more consistent with the Social Identity Theory than the Norms-Focused Theory^12^.

We aimed to further address this issue by analyzing potential modulatory influences of experimentally induced group membership on recipient’s brain activity in the UG, as measured by event-related potentials (ERP) and event-related oscillations (ERO) in the electroencephalogram. We experimentally induced ingroup and outgroup status and compared subjects’ electrocortical responses to extremely unequal, moderately unequal and equal offers as they interacted with ingroup or outgroup proposers in the UG. Behaviorally, the acceptance rates should decrease with the fairness level of the offers. In addition, as prior research has been broadly consistent with the Social Identity Theory, we predicted that acceptances rates should be higher when interacting with ingroup than with outgroup members.

In our ERP analyses, we focused on two components which reflect different stages of outcome-processing, the feedback related negativity (FRN) and the P3. The FRN is typically observed as a negative deflection in response to unfavorable compared to favorable action outcomes, peaking between 250–350 ms at frontocentral recording sites^15^. It reflects an early good versus no-good evaluation and is probably generated in the posterior portion of the medial frontal cortex^16^,^17^. Several studies have shown that the FRN can also be observed following unfair compared to fair offers in the UG or similar tasks^15^,^18^,^19^,^20^,^21^,^22^,^23^. Accordingly, we expected more negative FRN amplitudes in response to extremely and moderately unequal offers compared to equal offers in our study. Moreover, we predicted that this FRN effect would be modulated by ingroup/outgroup membership of the proposer. This assumption is based on recent studies showing that the FRN difference between unfair and fair offers is influenced by the social distance between proposers and recipients (e.g., being friends or strangers)^21^,^24^. According to Social Identity Theory, the salient social identity can determine cognitive focus as well as affective and behavioral responses^25^. Thus, recipients in the UG might shift their expectation and attention towards interactive partners, and expect an ingroup member to be more fair than an outgroup member. With higher fairness expectancies towards ingroup members, unfair offers by an ingroup proposer should result in stronger perceptions of fairness norm violations. As the FRN is sensitive to expectancy violations^26^,^27^,^28^, it should be especially pronounced for such unfair offers made by ingroup proposers.

The P3 is another ERP component that has frequently been found to be related to various aspects of outcome evaluation. It typically peaks about 250–500 ms post-stimulus onset and has a centro-parietal maximum. Some studies found that the P3 is sensitive to the magnitude of reward, with more positive amplitudes to a larger than to a smaller reward^16^,^20^. Other studies suggested that the P3 is also sensitive to reward valence, with more positive amplitude for positive than for negative reward^29^,^30^,^31^. Wu and colleagues found that the P3 is more positive to equal offers than to unequal offers, which might reflect differential distribution of attentional resources to offers with different affective/motivational significance^22^,^23^,^24^. In the present design and from the recipient’s perspective, the magnitude of reward co-varied with the valence of reward: a fair offer was also larger in magnitude than an unfair offer. Thus one might predict that, compared to extremely and moderately unequal offers, equal offers would elicit enhanced P3 responses. However, it was not clear whether and how the P3 would be modulated by group membership.

When analyzing ERP components of interest (the FRN, P3, etc.), their temporal overlap is often a troublesome problem. Principal components analysis (PCA) is a data reduction technique that can help to disentangle overlapping ERPs by providing a small set of statistically derived components which can explain the observed data^32^,^33^. Moreover, traditional ERP analysis assumes that the EEG response to relevant events is contained within a background of irrelevant neuroelectric noise, and that averaging several event-locked EEG time-traces can minimize this noise. However, a mount of evidence suggests that event-related changes in the magnitude and phase of the EEG signal across all frequencies may be relevant to information processing. Single-trial wavelet-based time-frequency analysis is a data decomposition method that can obtain a more thorough understanding of neuronal events^34^,^35^. Recent investigations have demonstrated that mid-frontal theta activity (about 4–8 Hz) is larger for negative feedback compared to positive feedback and may underlie the FRN effect in the EEG time-domain^34^,^36^. Mid-frontal theta-band oscillations are possibly generated in the anterior cingulate cortex (often also referred to as dorsal midcingulate cortex)^37^ and reflect the activity of performance monitoring systems in face of uncertainty^38^. In the present study, we therefore also used applied PCA to separate the FRN and P3, and time-frequency analysis to explore the frequency characteristics of feedback processing.

In sum, the current study aims to examine the influence of proposers’ ingroup/outgroup membership on recipients’ fairness considerations in the UG as indexed by acceptance/rejection rates, ERP (FRN and P3) responses and ERO (theta band) responses.

## Results

### Behavioral Results

#### Acceptance Rates

**Figure 1:**
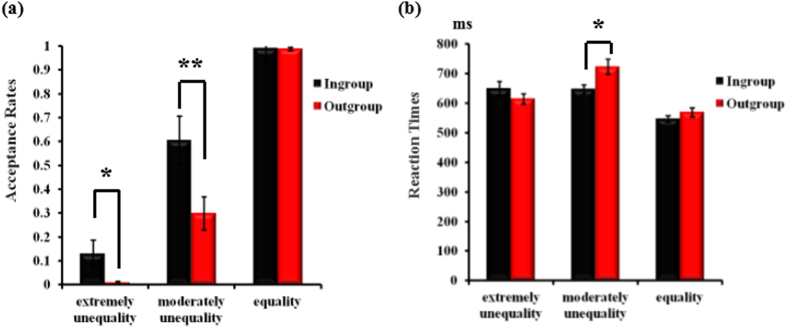
The acceptance rates and reaction times in the UG as a function of the offer type. Bars indicate standard error. Asterisks indicate significant effects (*p < 0.05, **p < 0.01).

The acceptance rates for different offers are presented in Fig. 1a. A 2 (group membership: ingroup vs. outgroup) × 3 (offer type: offer 1:9 vs. offer 3:7 vs. offer 5:5) repeated measures ANOVA revealed a significant main effect of offer type, *F*(2,30) = 122.11, *p* < 0.001, *η*_*p*_^2^ = 0.89, with higher acceptance rates for offer 5:5 (mean ± SE, 0.99 ± 0.01) than for offer 3:7 (mean ± SE, 0.45 ± 0.07, *p* < 0.001) and offer 1:9 (mean ± SE, 0.07 ± 0.03, *p* < 0.001), and for offer 3:7 than offer 1:9 (*p* < 0.001). The main effect of group membership was also significant, *F*(1,15) = 13.34, *p* = 0.002, *η*_*p*_^2^ = 0.47, with higher acceptance rates in intragroup interaction (mean ± SE, 0.58 ± 0.04) relative to intergroup interaction (mean ± SE, 0.43 ± 0.03). Importantly, the interaction between group membership and offer type was also significant, *F*(2,30) = 6.21, *p* = 0.006, *η*_*p*_^2^ = 0.29. Simple-effect tests showed that acceptance rates to offer 1:9 were significantly higher when interacting with ingroup proposers (mean ± SE, 0.13 ± 0.06) compared to outgroup proposers (mean ± SE, 0.01 ± 0.01), *F*(1,15) = 5.22, *p* = 0.037. In addition, the acceptance rates for offer 3:7 were significantly higher when interacting with ingroup (mean ± SE, 0.61 ± 0.10) than with outgroup proposers (mean ± SE, 0.30 ± 0.07), *F*(1,15) = 10.14, *p* = 0.006. For offer 5:5 there was no significant difference between proposer types, *F*(1,15) = 0.04, *p* = 0.841.

#### Reaction Times

The reaction times for different offers are presented in Fig. 1b. A 2 (group membership: ingroup vs. outgroup) × 3 (offer type: offer 1:9 vs. offer 3:7 vs. offer 5:5) repeated measures ANOVA revealed a significant main effect of offer type, *F*(2,30) = 33.90, *p* < 0.001, *η*_*p*_^2^ = 0.69, with longer reaction times to offer 3:7 (mean ± SE, 684.95 ± 15.79 ms) than to offer 5:5 (mean ± SE, 557.81 ± 11.66 ms, *p* < 0.001) and offer 1:9 (mean ± SE, 632.33 ± 16.93 ms, *p* < 0.01), and to offer 1:9 than offer 5:5 (*p* < 0.001). Moreover, the interaction between group membership and offer type was also significant, *F*(2,30) = 7.60, *p* = 0.002, *η*_*p*_^2^ = 0.37. Simple-effect tests showed that the reaction times to offer 3:7 was significantly higher when interacting with outgroup (mean ± SE, 723.16 ± 26.01 ms) compared to ingroup proposers (mean ± SE, 646.73 ± 14.97 ms), *F*(1,15) = 7.26, *p* = 0.017. There was no significant difference between the two proposers for offer 5:5, *F*(1,15) = 2.69, *p* = 0.122, nor for offer 1:9, *F*(1,15) = 2.29, *p* = 0.151.

#### Analysis of changes and stability in acceptance rates over time

Accordingly we analyzed changes in acceptance rates over time, we used the HLM6 software^39^ to separately conduct a hierarchical generalized linear modeling (HGLM) analysis of binary outcome for offer 1:9 and 3:7 from ingroup or outgroup members. The results revealed that participants’ reaction to offer 1:9 (*γ* = 0.00, *t* = 0.72, *p* = 0.485, odds ratio = 1.00) or 3:7 (*γ* = 0.00, *t* = −0.49, *p* = 0.634, odds ratio = 1.00) did not change across time when interacting with ingroup members. When interacting with outgroup members, participants more often accepted offer 3:7 (*γ* = 0.02, *t* = 2.62, *p* = 0.02, odds ratio = 1.02) with increasing trial count, but their reaction to offer 1:9 (*γ* = 0.00, *t* = 0.13, *p* = 0.897, odds ratio = 1.00) did not change over time.

### ERP Results: Temporospatial PCA factors

**Figure 2:**
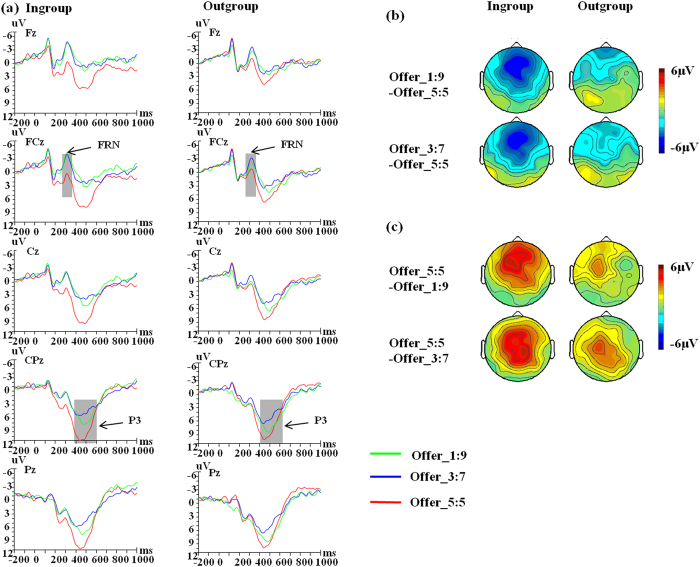
ERP responses and topograhic maps. (**a**) ERP responses time-locked to the onset of different offers at the midline Fz, FCz, Cz, CPz and Pz. The shaded 250–350, 400–600 ms time window was used for the calculation of the peak amplitudes of the FRN and P3 respectively. (**b**) Topographic maps for the FRN effects in the 250–350 ms time window. (**c**) Topographic maps for the P3 effects in the 400–600 ms time window.

**Figure 3:**
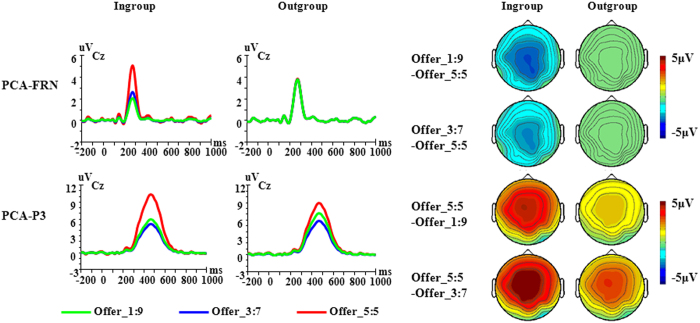
PCA results for different offers from ingroup/outgroup partner, as well as the corresponding scalp topographies. The waveforms of the PCA components were derived from the corresponding peak channel.

Figure 2 shows grand average ERP waveforms across the 16 participants. According to the research by Dien^32^,^33^, the most effective method for deciding which PCA factors to include in statistical analyses is to evaluate factors according to a priori knowledge about the ERP components relevant to the experimental design. Thus, based on the visual inspection of the waveforms, two factors were chosen for further statistical analyses, corresponding to the FRN and P3 in the grand average waveforms (Table 1). The waveforms for the two factors were reconstructed back into voltage space, and the FRN and P3 corresponding factors were quantified by the peak values at the peak channel for each condition (Fig. 3). Afterwards, a repeated measures 2 (group membership) × 3 (offer type) ANOVA was performed on each PCA factor.

### PCA factor corresponding to FRN component (TF4/SF1)

The main effect of offer type was significant, *F*(2,30) = 6.41, *p* = 0.005, *η*_*p*_^2^ = 0.30, suggesting that the PCA-FRN was more negative for offer 1:9 (mean ± SE, 2.89 ± 0.93 μV) and offer 3:7 (mean ± SE, 3.20 ± 1.13 μV) than for offer 5:5 (mean ± SE, 4.47 ± 1.05 μV), *p*s < 0.05. PCA-FRN responses to offer 3:7 and offer 1:9 did not differ, *p* > 0.05. The interaction between group membership and offer type was significant, *F*(2,30) = 5.48, *p* = 0.009, *η*_*p*_^2^ = 0.27, Post-hoc analysis indicated that the different offers from outgroup proposers did not influence the amplitude of the PCA-FRN, *F*(2,30) = 0.02, *p* = 0.978. The PCA-FRN did show differential responses to different offers from ingroup proposers, with offer 1:9 (mean ± SE, 2.06 ± 0.89 μV) and offer 3:7 (mean ± SE, 2.65 ± 1.08 μV) eliciting more negative amplitudes than offer 5:5 (mean ± SE, 5.08 ± 1.05 μV), *p*s < 0.01. Offer-wise contrasts between the ingroup and outgroup condition revealed no significant effects for offer 5:5, *F*(1,15) = 3.63, *p* = 0.076, and offer 3:7, *F*(1,15) = 2.66, *p* = 0.124. In contrast, for offer 1:9 we observed a significant difference between the ingroup and outgroup condition, *F*(1,15) = 10.73, *p* = 0.005, with the PCA-FRN being more negative when interacting with ingroup (mean ± SE, 2.06 ± 0.89 μV) compared to outgroup proposers (mean ± SE, 3.73 ± 1.04 μV).

In addition, we calculated the difference amplitudes between offer types (e.g. difference amplitudes for 1:9 minus 5:5 and for 3:7 minus 5:5), and then respectively performed a paired-samples *t*-tests. For the difference amplitudes for 1:9 minus 5:5, we found the difference amplitudes were more negative when interacting with ingroup proposers (mean ± SE, −3.02 ± 0.57 μV) than with outgroup proposers (mean ± SE, −0.15 ± 0.85 μV), *t*(15) = −3.15, *p* = 0.007. For the difference amplitudes for 3:7 minus 5:5, there was no significant difference between two interactive conditions, *t*(15) = −2.07, *p* = 0.056.

### PCA factor corresponding to P3 component (TF1/SF1)

The main effect of offer type was significant, *F*(2,30) = 12.81, *p* < 0.001, *η*_*p*_^2^ = 0.46, such that the PCA-P3 were more positive for offer 5:5 (mean ± SE, 9.64 ± 1.05 μV) than for offer 3:7 (mean ± SE, 5.51 ± 1.20 μV) and offer 1:9 (mean ± SE, 6.59 ± 1.25 μV), *p*s < 0.05. PCA-P3 responses to offer 3:7 and offer 1:9 did not differ, *p* = 0.609. The interaction between group membership and offer type was significant, *F*(2,30) = 3.34, *p* = 0.049, *η*_*p*_^2^ = 0.18. Post-hoc analysis showed that the PCA-P3 showed differential responses to different offers from ingroup proposers, *F*(2,30) = 16.91, *p* < 0.001, with offer 5:5 (mean ± SE, 10.29 ± 1.25 μV) eliciting more positive PCA-P3 amplitudes than offer 3:7 (mean ± SE, 5.15 ± 1.25 μV) and offer 1:9 (mean ± SE, 5.95 ± 1.19 μV), *p*s < 0.01. Offer type also influenced the P3 responses when interacting with outgroup proposers, *F*(2,30) = 4.54, *p* = 0.019, with offer 5:5 (mean ± SE, 8.99 ± 1.04 μV) eliciting more positive PCA-P3 than offer 3:7 (mean ± SE, 5.88 ± 1.31 μV), *p* = 0.038. We did not observe any difference between the ingroup and outgroup condition when analyzing the offer types separately, all *p* > 0.154.

Moreover, we calculated the difference amplitudes between offer types (e.g. difference amplitudes for 1:9 minus 5:5 and for 3:7 minus 5:5), and then respectively performed a paired-samples *t*-tests. For the difference amplitudes for 1:9 minus 5:5, we found the difference amplitudes were more negative when interacting with ingroup proposers (mean ± SE, −4.34 ± 1.18 μV) than outgroup proposers (mean ± SE, −1.76 ± 0.95 μV), *t*(15) = −2.62, *p* = 0.019. For the difference amplitudes for 3:7 minus 5:5, there was no significant difference between two interactive conditions, *t*(15) = −1.85, *p* = 0.084.

### ERO Results

**Figure 4:**
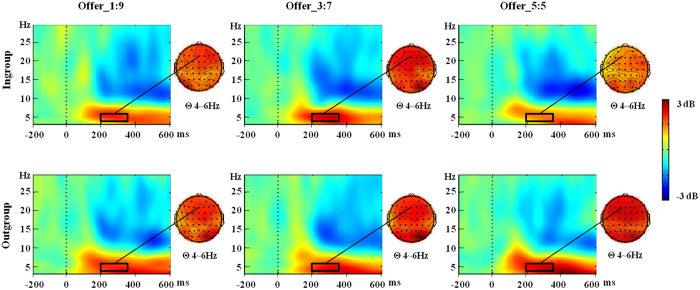
ERSP results for different offers from ingroup/outgroup partner at Fz. Dark rectangles mark the time/frequency window used in the statistical analysis and the corresponding scalp topographies.

Figure 4 shows grand average ERO plots across the 16 participants at Fz. A 2 (group membership: ingroup vs. outgroup) × 3 (offer type: offer 1:9 vs. offer 3:7 vs. offer 5:5) × 5 (electrode: Fz vs. FCz vs. Cz vs. CPz vs. Pz) repeated measures ANOVA was conducted on the mean amplitudes of theta power. There was only a significant interaction of group membership x offer type x electrode, *F*(8,120) = 3.15, *p* = 0.027, *η*_*p*_^2^ = 0.17. A further simple test found the theta power differentially responded to different offers from ingroup proposers over electrode Fz, *F*(2,30) = 5.29, *p* = 0.011, with offer 3:7 (mean ± SE, 2.40 ± 0.45 dB) eliciting larger theta power than offer 5:5 (mean ± SE, 1.58 ± 0.45 dB), *p* = 0.041. In contrast, we observed no significant theta power modulation by offer type in the outgroup condition over five electrodes, all *p* > 0.206. When directly comparing the ingroup and outgroup condition separately for each offer over five electrodes, group membership did affect theta power for 5:5 offers at electrode Fz, *F*(1,15) = 4.91, *p* = 0.043, and FCz, *F*(1,15) = 5.37, *p* = 0.035. At these two electrodes, theta power was larger when interacting with outgroup proposers (mean ± SE, Fz: 2.58 ± 0.34 dB, FCz: 2.69 ± 0.36 dB) than with ingroup proposers (mean ± SE, Fz: 1.50 ± 0.47 dB, FCz: 1.58 ± 0.45 dB). However, group membership did not have a significant effect for offer 1:9 and offer 3:7 over five electrodes, all *p* > 0.244.

## Discussion

In the study presented, we aimed to investigate how this factor affects neurophysiological correlates of fairness considerations in the prominent UG. We found that participants were more likely to accept unequal offers from ingroup compared to outgroup members. In line with previous studies, this result supports the Social Identity Theory, suggesting that people are more tolerant to social norm violating behaviors executed by ingroup members. Moreover, group membership can modulate participants’ reaction to unequal offers over time. Participants’ reactions to unequal offers did not change across time in the ingroup interaction, while they do accept more unequal offer from outgroup members over time. In a repeated game, one would expect that reactions to unfairness change over time, based on partner’s behavior, and participants’ expectations would supersede the group conformity norm. However, the stable reaction to unequal offer from ingroup members indicates that participants might follow the group conformity norm in all interactions in order to maintaining and stabilizing the ingroup integrity. In contrast, the increasing tolerance to unequal offers from outgroup over time might support the expectation norm. As the interaction proceeds, participants might feel that the moderately unequal offers are exceeding their initial expectations about outgroup members, and consider these moderately unequal offers as acceptable during the context of group competition. So, these results indicates participants may adopt the group conformity norm towards ingroup members, and the expectations norm to outgroup members in our study.

Our results indicate that ingroup/outgroup membership has a substantial influence on earlier and later processes of fairness evaluation as reflected by the well-known FRN and P3, respectively. Probably most strikingly, the differentiation between fair and unfair offers in the FRN amplitude was most pronounced when offers were made by an ingroup proposer. In contrast, this effect was much smaller and statistically insignificant when offers were made by an outgroup proposer. This modulatory effect is likely to be driven by group-based fairness expectations. Previous studies have repeatedly shown that the FRN is sensitive to expectancy violations, with FRN amplitude being especially high (i.e., more negative) for events that are worse than expected^27^,^31^,^40^. In our study, the experimentally induced group membership has probably led to the expectation that ingroup proposers will behave in a more or less fair way (i.e., make a 5:5 offer). Unfair offers by these proposers violate this expectation and, consequently, lead to more negative FRN amplitudes. For the outgroup proposers unfair offers are presumably more or less expected and therefore do not evoke a pronounced FRN. Interestingly and in line with prior research^7^,^8^, unfair offers were also more often accepted when made by ingroup compared to outgroup proposers. Thus, even though such offers appear to be evaluated as worse than expected, the individual rather tends to accept them. As outlined above, this may also indicate that the early negative evaluation (as reflected by the FRN), which itself results from the perception of a norm-violation, is overcome to behave in a norm-conform way. Such mechanism could also play a crucial role in maintaining and stabilizing the ingroup integrity when threatened by norm violations of single group members.

Our FRN results may also be interpreted in the light of two previous ERP studies which have shown that the FRN differentiation between fair and unfair proposals also differs, depending on whether proposer and receiver are friends or strangers^21^,^24^. However, results of these two studies were contradictory. Very similar to our findings, Wu *et al*. reported a FRN by offer modulation for friends but not for strangers^24^. In contrast, Campanhã *et al*. found a more negative FRN for unfair compared to fair offers made by strangers, whereas this effect was reversed for offers made by friends^21^. These inconsistencies between studies may result from different paradigms (the dictator game vs. the ultimatum game), cultural differences (Chinese vs. Brazilian samples) and different degrees of anonymity. First, the economic games used in the studies differ. The dictator game is a modification of the UG in which the responder cannot refuse the offer and money is always distributed as proposed. Previous research has shown that recipients expect higher outcomes when they have more retaliatory power^41^. In close relationships, fairness concerns are less important for friends than for strangers as their friends would be less worried about unfair offers being rejected. Thus, when playing with strangers, social expectations change dramatically from the dictator game to ultimatum game but expectations change little across games when playing with friends. Second, another important difference between the two studies is the cultural differences. People’ responses to norm violations are highly variable across cultures. While punishment of low offers in economic games is common in some cultures, in other cultures so-called ‘antisocial’ punishment of generous offers frequently occurs^42^. The Chinese may adopt different norms towards friends than their Western counterparts, and they might expect reciprocity and benefits from friends to a greater degree^43^. Thus, the FRN responses to offers from friends in Wu *et al*. may be attributed to the participants’ more automatic responses to the violation of social norms associated with friendship. The third alternative possibility is related to the difference of anonymity. Wu *et al*. used two friends (and two strangers) to pair with one participant such that participants were uncertain about the identity of their partners. In contrast, Campanhã *et al*. used only one friend (and one stranger) to pair with one participant, making it clear with whom the participant was interacting. Although extensive research has demonstrated that anonymity is a significant factor influencing human behavior, a recent research found that degree of anonymity did not modulate the FRN patterns in response to unfairness^44^. Hence, the discrepancies between the two studies are more likely due to the different paradigms used and the cultural difference. Future studies may further investigate this issue by directly comparing samples from different cultural populations. More importantly, as the friendship contained multiple elements such as sympathy, and trustfulness, our experimental comparison, i.e., ingroup strangers versus outgroup strangers, is likely to be treated very differently. For example, people might accept unfair offer from friends to protect their close friendship, and divide the monetary gain after completing the experiment. In contrast, group membership would automatically vanish after completing the experiments. Hence, our results extend existing research, and found that the early evaluative mechanism indexed by the FRN is sensitive to the fairness of offers and that this effect is modulated by ingroup/outgroup relation between receivers and proposers.

Besides disadvantageous unequal offers (i.e. offer 1:9 or 3:7) some researchers have also investigated the role of advantageous unequal offers (i.e. Offer 9:1 or 7:3). While chimpanzees show no evidence of sensitivity to advantageous inequality^45^, advantageous inequality aversion represents a unique feature of human behavior. An ERP study let participants act as recipients, receiving either disadvantageous unequal, equal, or advantageous unequal offers in the UG, and found the advantageous unequal offers also elicited more negative-going FRN amplitudes compared to equal offers. This finding indicates that advantageous unequal offers are also perceived as violation of the equity rule^32^. A recent functional magnetic resonance imaging study found that people have strong preferences for fairness in both disadvantageous and advantageous inequality situations, and both types of inequality activated the putmamen, orbitotrontal cortex, and insula^44^. More importantly, people who are more averse to advantageous inequality had enhanced activity in putamen and less functional connectivity between putamen and both orbitofrontal cortex and anterior insula. People who are more averse to disadvantageous inequality show increased activity in amygdala and less functional connectivity between amygdala and anterior cingulate cortex. These results indicate that both types of inequality are processed by similar brain areas, yet modulated by different neural pathways^44^.

In addition, we also observed a P3 modulation by group membership. In detail and similar to our FRN results, the differentiation between offer types in the P3 amplitude was more pronounced when offers were made by ingroup compared to outgroup proposers. Thus, it may be inferred that also later mechanisms of fairness evaluation of others’ behavior are more strongly engaged when interacting with an ingroup member. In particular, previous research has indicated that P3 amplitude is closely linked to the motivational salience of a certain event^27^,^28^. Therefore, our results indicate that the evaluation of the motivational value of a particular offer in the UG is more differentiated when this offer is made by an interaction partner belonging to the same social group.

Moreover, we also observed a theta band power modulation by group membership. In detail and similar to our FRN results, the differentiation between offer types in the theta band power was more pronounced when offers were made by ingroup compared to outgroup proposers. Previous research has indicated that theta oscillations may (at least partially) underlie the FRN effect in the EEG time-domain and reflect the activity of performance monitoring system in face of uncertainty^36^,^37^. Therefore, our results indicate that the early evaluative mechanism indexed by the theta band is also more differentiated when this offer is made by an interaction partner belonging to the same social group.

Altogether, our findings show that ingroup/outgroup membership strongly influences early as well as later neural mechanisms of fairness evaluation in the UG. In particular, these mechanisms appear to be much more engaged when interacting with a member of one’s own social group. Presumably, the observed effects, especially for the FRN and theta power, are mainly driven by norm-based expectations (and the violation thereof) with regard to social-rule conformity. Interestingly, when comparing behavioral and EEG results our study also points to a principle of “grin and bear it” in interpersonal bargaining. That is, while unfair offers might be quickly evaluated as negative when made by an ingroup compared to an outgroup member, individuals appear to overcome this negative evaluation, accepting unfair offers by ingroup members more often, maybe for the sake of group integrity. It is up to future research to uncover the cognitive-affective mechanisms involved in this group securing process.

## Methods

### Participants

Sixteen undergraduate and graduate students (8 females and 8 males, age range 19–25 years, mean age 21.3 years) without prior history of neurological or psychiatric dysfunction completed the experiment. All of them were right-handed and had normal or corrected-to-normal vision. Informed written consent was obtained from all participants prior to the experiment. The experimental protocol was approved by the local Ethics Committee (Tianjin Normal University), and was in compliance with the ethical procedures of the American Psychological Association (APA).

All the participants were paid 20 Chinese yuan (about $ 3) as basic payment and were informed that additional monetary rewards would be paid according to their performance in the task, although in the end all participants were paid extra 10 yuan on top of the basic payment. Four graduate students (one pair of females and one pair of males), who were strangers to the EEG participants, were recruited as confederates. To exclude possible influence of sex on fairness consideration, each participant was grouped with a pair of same sex strangers, who played the role of proposers in the UG.

### Design and procedures

After arriving at the laboratory, each participant was asked to complete a picture selection task in which he/she took one of two photographs (upside down) of same sex confederates. Before taking the photograph, participants were informed that the two confederates had been assigned randomly either to the red group or the blue group, and that he/she and the selected confederate would belong to the same group. In other words, the selected confederate would be the ingroup member, while the unselected confederate would be the outgroup member for each participant. The in-group category (i.e. the red or blue group) was counterbalanced between subjects. After completing this task, the subject met the two same sex confederates and all of them were asked to stand against the wall and a picture of each person was taken using a digital camera. Finally, each of them was asked to wear a badge of the respective color.

The participant and the two confederates were then told that they would sit in separate rooms to finish a task together through a computer network, and that the participant would play as a recipient and the others would be proposers. He/She was also informed about the rules of the UG and that he/she would interact respectively with the ingroup member and outgroup member in two separate UG blocks. Moreover, the subject was told that in order to reduce the difficulty and the range of offers, proposers have to make a choice from 1:9 offer (1 yuan for the receiver, 9 yuan for the proposer), 3:7 offer or 5:5 offer. Participant was asked to press a button with the index finger of his/her left or right hand to accept or reject the offer, respectively. Finally, given that group affiliation is often strongest in contexts involving competition between groups, the participant was also informed that the experimenter would determine which group is victorious according to the average earnings of each group after all interactions. This manipulation was included as an attempt to further differentiate feelings toward ingroup and outgroup partners, and was just a verbal feedback that did not lead to any punishment or reward.

**Figure 5:**
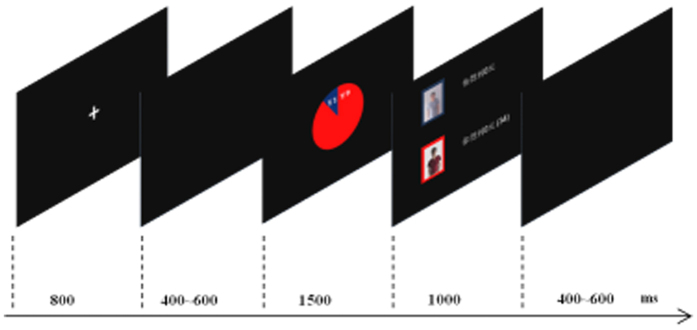
Timeline of a single trial in the Ultimatum game.

At the beginning of each block, the subject was informed whether the proposer was an ingroup or outgroup member. Each trial started with the presentation of a fixation cross for 800 ms, followed by a black screen for 400–600 ms. (see Fig. 5) Afterwards, a divided color pie was presented that indicated the amount of the offer (1500 ms). The subject was asked to make the “accept” or “reject” decision within the duration as quickly as possible. This was followed by a feedback, in which a photo of the participant and the pseudo-proposer were presented for 1000 ms together with the amount of money received by each one of them and the cumulative amount of the participant’s winnings. Finally, a 400–600 ms black screen was presented.

The participant was seated comfortably about 1 m in front of a computer screen. The UG comprised two blocks (ingroup vs. outgroup) of 90 trials each. Block-order was counterbalanced between subjects. Unknown to the participants, each of the three offer types was presented 30 times per block in a pseudo-randomized order with the restriction that no more than 3 consecutive trials were of the same offer type. A practice block of 10 trials was administered before the formal test, in which the proposer was alternating between ingroup and outgroup member.

### EEG Recording and Analysis

We measured brain electric activity from 64 channels with the averaged bilateral mastoid reference and a forehead-ground, using a modified 10–20 system electrode cap (Neuroscan Inc.). The vertical EOG activity was recorded with electrodes placed above and below the left eye, and horizontal EOG activity was recorded with electrodes placed at the outer canthi of both eyes. All electrode sites were cleaned with alcohol and inter-electrode impedance was maintained below 10 kΩ. Bio-signals were amplified at a band-pass from 0.05 to 100 Hz and continuously sampled at 1000 Hz/channel. Eye blink artifacts were removed automatically using Scan software (Neruoscan Inc.). Data were segmented into offer-locked epochs of 1000 ms with a 200 ms pre-stimulus baseline window. All segments with EEG voltages exceeding ±100 μV were excluded from further analysis. Each offer condition contained at least 26 trials. After averaging, the mean amplitude of the baseline time window was subtracted from each data-point. The FRN and P3 were quantified using temporospatial PCA following the two-step procedure. PCA was conducted with the EP Toolkit (v2.45)^32^ for MATLAB, using the covariance matrix and Kaiser Normalization. Following recently published sets of guidelines for applying PCA to ERP datasets^33^,^46^, a temporal PCA was performed on the data first to capture variance across time points. Promax rotation was used, and fifteen temporal factors were extracted based on the resulting scree plot^47^. Following this, a spatial PCA was performed on each temporal factor and Infomax was used to rotate to independence in the spatial domain. Based on the averaged scree plot for fifteen temporal factors, three spatial factors were extracted, yielding 45 unique factors combinations. Based on the visual inspection of the waveforms, two factors were chosen for further statistical analyses, corresponding to the FRN and P3 in the grand average waveforms (Table 1).

### Time-frequency Analysis

Time-frequency analysis was performed using a complex Morlet wavelet transform as implemented by the EEGLAB toolbox (v13.4.4b) for MATLAB. The wavelet transform is a multiresolution analysis technique that provides a good compromise between time and frequency resolution^48^. The complex Morlet wavelet *w*(*t, f*_0_) has a Gaussian distribution in the time (*σ*_*t*_) and frequency (*σ*_*f*_) domains around the center frequency *f*_0_. All analyses were performed using custom in-house routines written using EEGLAB running under MATLAB. The epoching of continuous EEG files and artifact-related processing was performed with Scan software (Neruoscan Inc.). Single-trial epochs were extracted from −1000 to 2000 ms relative to stimulus, and the raw EEG was down-sampled to 500 Hz. A Morlet-based wavelet transform procedure as implemented in EEGLAB (v13.4.4b) was employed (3 through 35 Hz) in order to provide a continuous estimate of the power of a given frequency between −1000 and 2000 ms^35^.

Event-related spectral perturbations (ERSP) were computed on the wavelet-transformed epochs for each condition at each time point and wavelet frequency to yield time-frequency maps^49^. Power values were normalized with respect to a −400 to −200 ms pre-stimulus baseline and converted to decibels [10 × log(μV^2^)]. Based on the previous studies on theta frequency and the results of permutation test implemented in the statcond function of EEGLAB toolbox, ERSPs in the range of 4–6 Hz during 200–350 ms were averaged for further statistical analysis. For statistical analyses, we focused on the midline electrode sites Fz, FCz, Cz, CPz, and Pz. The theta band power participated to repeated measures ANOVAs with three within-participant factors: group membership (ingroup vs. outgroup), offer type (offer 1:9 vs. offer 3:7 vs. offer 5:5), and electrode (Fz, FCz, Cz, CPz, and Pz). Only significant effects were reported. P- values of all main and interaction effects were corrected using the Greenhouse-Geisser method for repeated-measures effects. The Bonferroni correction was used for multiple comparisons. Because the topographic distributions of power exhibited a frontal peak, the ERSP results at Fz was only illustrated.

## Additional Information

**How to cite this article**: Wang, Y. *et al*. Ingroup/outgroup membership modulates fairness consideration: neural signatures from ERPs and EEG oscillations. *Sci. Rep.* **7**, 39827; doi: 10.1038/srep39827 (2017).

**Publisher's note:** Springer Nature remains neutral with regard to jurisdictional claims in published maps and institutional affiliations.

## Figures and Tables

**Table 1:** Temporospatial PCA factors selected for data analysis.

Corresponding ERP component	PCA factors	Variance explained (%)	Peak latency (ms)	Peak channel
FRN	TF4/SF1	4.56	271	Cz
P3	TF1/SF1	19.4	466	Cz

SF = spatial factor, TF = temporal factor.
